# Low incidence of ABL-class and JAK-STAT signaling pathway alterations in uniformly treated pediatric and adult B-cell acute lymphoblastic leukemia patients using MRD risk-directed approach – a population-based study

**DOI:** 10.1186/s12885-020-07781-6

**Published:** 2021-03-29

**Authors:** Rimvydas Norvilas, Vaidas Dirse, Ruta Semaskeviciene, Orinta Mickeviciute, Egle Gineikiene, Mindaugas Stoskus, Goda Vaitkeviciene, Jelena Rascon, Laimonas Griskevicius

**Affiliations:** 1 Hematology, Oncology and Transfusion Medicine Center, Vilnius University Hospital Santaros Klinikos, Vilnius, Lithuania; 2 Department of Experimental, Preventive and Clinical Medicine, State Research Institute Centre for Innovative Medicine, Vilnius, Lithuania; 3 Institute of Clinical Medicine, Vilnius University, Vilnius, Lithuania; 4 Center for Pediatric Oncology and Hematology, Vilnius University Hospital Santaros Klinikos, Vilnius, Lithuania

**Keywords:** B-ALL, ABL-class, JAK-STAT, RNA-Seq

## Abstract

**Background:**

ABL-class and JAK-STAT signaling pathway activating alterations have been associated with both a poor post-induction minimal residual disease (MRD) response and an inferior outcome in B-cell acute lymphoblastic leukemia (B-ALL). However, in most of the studies patients received non-uniform treatment.

**Methods:**

We performed a population-based analysis of 160 (122 pediatric and 38 adult) Lithuanian *BCR*-*ABL1*-negative B-ALL patients who had been uniformly treated according to MRD-directed NOPHO ALL-2008 protocol. Targeted RNA sequencing and FISH analysis were performed in cases without canonical B-ALL genomic alterations (high hyperdiploids and low hypodiploids included).

**Results:**

We identified ABL-class fusions in 3/160 (1.9%) B-ALL patients, and exclusively in adults (*p* = 0.003). JAK-STAT pathway fusions were present in 4/160 (2.5%) cases. Of note, *P2RY8-CRLF2* fusion was absent in both pediatric and adult B-ALL cases. Patients with ABL-class or JAK-STAT pathway fusions had a poor MRD response and were assigned to the higher risk groups, and had an inferior event-free survival (EFS) / overall survival (OS) compared to patients without these fusions. In a multivariate analysis, positivity for ABL-class and JAK-STAT fusions was a risk factor for worse EFS (*p* = 0.046) but not for OS (*p* = 0.278) in adults.

**Conclusions:**

We report a low overall frequency of ABL-class and JAK-STAT fusions and the absence of *P2RY8-CRLF2* gene fusion in the Lithuanian *BCR-ABL1* negative B-ALL cohort. Future (larger) studies are warranted to confirm an inferior event-free survival of ABL-class/JAK-STAT fusion-positive adult patients in MRD-directed protocols.

**Supplementary Information:**

The online version contains supplementary material available at 10.1186/s12885-020-07781-6.

## Background

B-cell acute lymphoblastic leukemia (B-ALL) is an aggressive and genetically heterogeneous disease which mostly affects children and young adults [1]. A number of genomic alterations has been associated with the B-ALL outcome resulting in risk-adapted treatment protocols [2]. Approximately one-third of both pediatric and adult B-ALL cases do not harbor any canonical genomic alterations [3]. Gene expression profiling (GEP) of patients negative for canonical B-ALL alterations identified a subgroup of cases characterized by a similar expression profile to those harboring t(9;22) (q34;q11)/*BCR-ABL1* (Philadelphia chromosome-like (Ph-like) or *BCR-ABL1*-like B-ALL) [4, 5].

Whole transcriptome/exome sequencing and fluorescence in situ hybridization (FISH) analysis of Ph-like B-ALL samples revealed high frequency of genomic alterations disrupting the normal function of kinase and cytokine receptor signaling pathways of which ABL-class kinase and JAK-STAT signaling pathway genes were the most commonly affected [6]. ABL-class gene and JAK-STAT pathway alterations are associated with high post-induction MRD levels, an increased relapse rate and inferior outcome. Of note, some of these gene fusions can be successfully targeted using small-molecule inhibitors [7–12]. The identification of a Ph-like B-ALL signature is not standardized and both ABL-class and JAK-STAT fusions can also be found outside of the Ph-like B-ALL group [13–16]. High expression levels of the *CRLF2* gene and *IKZF1* gene deletions are also common in the Ph-like B-ALL subgroup resulting in a worse outcome in some series [5, 17].

In most studies, a Ph-like B-ALL was clinically characterized as a high-risk group with inferior overall survival in intensively treated children and adults [18–20]. In contrast, Ph-like B-ALL children did not have inferior survival when a minimal residual disease (MRD)-directed treatment of Total Therapy XV protocol was used [21, 22]. Whether MRD-directed treatment can also improve the negative prognosis of Ph-like B-ALL adult patients remains to be defined.

In this study we performed a comparative genomic and clinical data analysis of pediatric and adult B-ALL patients to characterize the incidence of ABL-class and JAK-STAT signaling pathway activating alterations in the Lithuanian population and to determine their clinical significance in a MRD-risk directed treatment setting.

## Methods

### Patients and samples

From July 2008 to December 2017, pediatric (1─17 year-olds) and adult (18─45 year-olds) B-ALL patients who had been diagnosed with *BCR*-*ABL1*-negative B-ALL in Lithuania and enrolled into a NOPHO ALL-2008 clinical trial [2] were included. In addition, eleven 46─65 year-old adult patients were treated according to a NOPHO ALL-2008 protocol with a chemotherapy dose modification and outside of the clinical trial. The patients’ data were collected retrospectively.

The diagnosis of ALL was based on a bone marrow biopsy or aspirate showing ≥20% leukemic blasts. The protocol specific genomic analysis was limited to canonical B-ALL genomic alterations – t(12;21)/*ETV6-RUNX1*, t(1;19)/*TCF3-PBX1*, 11q23/*KMT2A* gene rearrangements, iAMP21, dic(9;20), high hyperdiploidy (51–67 chromosomes) and low hypodiploidy (31–39 chromosomes). Cases without canonical B-ALL translocations were selected for RNA sequencing. Patients with ploidy shifts (high hyperdiploidy or low hypodiploidy) were also selected for RNA sequencing as they could harbor additional kinase and cytokine receptor signaling pathway alterations [23, 24].

Patients were stratified to the three risk groups defined in the NOPHO ALL-2008 protocol detailed elsewhere [2]. Stratifying factors were white blood cell count, immunophenotype and cytogenetic markers at diagnosis and treatment response defined as MRD. High genetic risk was assigned if *KMT2A* gene rearrangements, iAMP21, dic(9;20) or low hypodiploidy were present. The MRD analysis was performed on days 15, 29 and 79 (only for standard risk (SR) and intermediate risk (IR) patients) or after each block (in high-risk (HR) arm until MRD-negativity). If MRD was ≥0.1% on day 79 (SR and IR) or after block B1 (HR), patients were referred to an allogeneic hematopoietic stem cell transplant (SCT) after receiving at least one additional block of therapy and having MRD below < 0.1% (optimally negative) (HR-SCT arm).

The study was conducted in accordance with the Declaration of Helsinki, and the protocol was approved by the Vilnius Regional Bioethics Committee. The patients provided written informed consent. The waiver for obtaining written informed consent from patients who could not be reached despite our best efforts was approved by the Vilnius Regional Bioethics Committee according to national regulations.

### RNA purification and targeted RNA-sequencing

Total RNA was purified from fresh bone marrow samples at the time of diagnosis using silica-membrane-based purification protocols and stored at − 80 °C. All bone marrow samples had blast counts ≥70%. Before RNA sequencing (RNA-Seq), purified RNA concentration and quality was assessed using NanoDrop2000 spectrophotometer.

RNA-Seq libraries were constructed using the Illumina TruSight Pan-Cancer sequencing kit (Illumina, San Diego, CA, USA), following the manufacturer’s protocol and recommendations. The libraries were sequenced using Illumina MiSeq genome analyzer (Illumina). A total of 1385 cancer-related genes were analyzed including frequently mutated genes of ABL-class (*ABL1, ABL2, PDGFRB, CSF1R*) and JAK-STAT (*JAK1, JAK2, JAK3, CRLF2*) signaling pathways. At least 4 million paired-end reads were obtained for each sample. NGS data analysis was performed using the Illumina BaseSpace Informatics Suite (Illumina). TopHat/STAR aligners and a Manta fusion caller were used to detect novel and recurrent gene fusions. At least one partner gene was required to detect novel gene fusion. Only high-confidence fusions were called that met threshold filters: split and paired unique reads (≥3), fusion contig align (≥16 bp in length), coverage after fusion (≥100 bp), break end homology (≤10 bp).

Additional gene mutation analysis from RNA-Seq data was performed using the GATK pipeline and Isaac Variant Caller 2.3. Point mutations and small indels were called if they had an allele frequency ≥ 5% and a population frequency < 0.01, were negative for known SNP’s and were previously described in genomic variant databases (COSMIC, ClinVar, OMIM, HGMD).

Prior to the study, the RNA-Seq method was first validated using thirteen B-ALL control samples with previously identified gene fusions (*BCR-ABL1 n* = 5, *KMT2A* gene rearrangements n = 5, *TCF3-PBX1 n* = 2, *ETV6-RUNX1 n* = 1). All in-frame gene fusions were identified, therefore the RNA-Seq method was used for B-ALL study patients.

### FISH, PCR and SNP Array data analysis of B-ALL fusions

Due to technical limitations of RNA-Seq, additional FISH analysis of *CRLF2* gene (*CRLF2* (Xp22/Yp11) Break / IGH Fusion,TC; Leica Biosystems, Wetzlar, Germany) was performed to identify *P2RY8-CRLF2* and *IGH-CRLF2* gene fusions. FISH analysis for *ABL1* (SPEC *ABL1* Dual Color Break Apart Probe; ZytoVision, Bremerhaven, Germany), *ABL2* (SPEC *ABL2* Dual Color Break Apart Probe; ZytoLight), *JAK2* (*JAK2* (9p24) Break; Leica Biosystems) and *PDGFRB* (*PDGFRB* (5q32) Break; Leica Biosystems) genes were used to confirm respective gene fusions detected by the RNA-Seq method. FISH analysis was performed according to manufacturer protocols.

Standard RT-PCR followed by a gel electrophoresis and Sanger sequencing were used to confirm in-frame fusions that were not confirmed with an additional FISH analysis. Fusion specific PCR primers were constructed for each case (Additional file 1: Table S1).

*P2RY8-CRLF2* fusion is a result a of 320 Kb size deletion of the PAR1 region in the short arm of either X or Y chromosomes. We performed SNP Array data analysis for detection of PAR1 region deletions in order to confirm the absence of *P2RY8-CRLF2* gene fusion not detected by RNA-Seq or FISH methods in our cohort. The SNP-Array method was based on the Infinium HD whole-genome genotyping assay with the HumanCytoSNP-12 BeadChip (Illumina Inc., San Diego, CA), which covers the entire genome with an average spacing of 9.6 kb. This coverage allows an average resolution of 31 kb which was used for detection of PAR1 region deletion.

### Statistical analysis

Differences in the prevalence of parameters between the groups were determined using the Mann-Whitney test or Independent-Samples T-test for continuous variables depending on their distributions. For categorical analyses, either a Chi-square or a Fisher exact test was used. Univariate and multivariate Cox proportional hazard regression models were used to evaluate the effect of non-canonical B-ALL genomic alterations on survival. The Kaplan-Meier method was used to estimate the time to event distributions (overall survival and event-free survival), the Log-Rank test was used to compare differences in survival curves. The overall survival (OS) was computed from the date of diagnosis until the date of death or last known follow-up date. The event-free survival (EFS) was defined as the time from diagnosis to the event of resistant disease, relapse, induction death, death in remission, second malignancy or date of the last follow-up if a patient had no events. A *p*-value < 0.05 was considered to indicate statistical significance. Statistical analyses were performed using SPSS software version 20.

## Results

### Study population

Overall, one hundred and sixty 1–65 year-old patients representing over 95% of the *BCR-ABL1* negative B-ALL patient population in Lithuania during the July 2008 – December 2017 period were included into this study (Table 1). One hundred and twenty-two (76.3%) patients were younger than 18 years of age. The majority of pediatric patients (59.5%) were stratified to the NOPHO ALL-2008 standard risk treatment arm. In contrast, more than half of the adult patients were stratified either to the intermediate (44.7%) or high risk/high risk SCT disease (29.0%).

**Table 1:** Clinical characteristics of B-ALL patients

	1─17 yo	18─65 yo	All	***p***-Value
**Age groups**				
**Number of patients**	122 (76.3%)	38 (23.7%)	160 (100%)	
**Median age (range)**	4 (1–17)	32 (18–65)	5 (1–65)	
**Sex:**				
**Male**	69 (56.6%)	15 (39.5%)	84 (52.5%)	0.093
**Female**	53 (43.4%)	23 (60.5%)	76 (47.5%)	
**Risk group:**				
**SR**	72 (59.5%)	7 (18.4%)	79 (49.7%)	< 0.001
**IR**	38 (31.4%)	17 (44.7%)	55 (34.6%)	
**HR**	5 (4.1%)	6 (15.8%)	11 (6.9%)	
**HR-SCT**	3 (2.5%)	5 (13.2%)	8 (5%)	
**Induction failure**	3 (2.5%)	3 (7.9%)	6 (3.8%)	
**Not risk grouped**	1	0	1	
**Induction:**				
**Prednisolone**	113 (92.6%)	30 (78.9%)	143 (89.4%)	0.030
**Dexamethasone**	9 (7.4%)	8 (21.1%)	17 (10.6%)	
**WBC (× 10**^**9**^**/l):**				
**< 100**	113 (92.6%)	30 (78.9%)	143 (89.4%)	0.030
**≥ 100**	9 (7.4%)	8 (21.1%)	17 (10.6%)	
**WBC median (range)**	14 (1–641)	10.5 (0.9–481.9)	13.5 (0.9–641)	0.672
**Hgb (g/l) median (range)**	83 (24–140)	90.5 (52–142)	84 (24–142)	0.117
**Platelets median (range)**	62 (0–457)	37 (5–320)	54.5 (0–457)	0.075
**CNS Status:**				
**CNS1**	104 (85.2%)	33 (86.8%)	137 (85.6%)	0.281
**CNS2**	14 (11.5%)	2 (5.3%)	16 (10%)	
**CNS3**	4 (3.3%)	3 (7.9%)	7 (4.4%)	

*SR* Standard risk, *IR* Intermediate risk, *HR* High risk, *HR-SCT* High risk–stem cell transplant groups, *WBC* White blood cells, *HgB* Hemoglobin, *CNS* central nervous system

### Genomic analysis

#### Gene fusion analysis

**Fig. 1:**
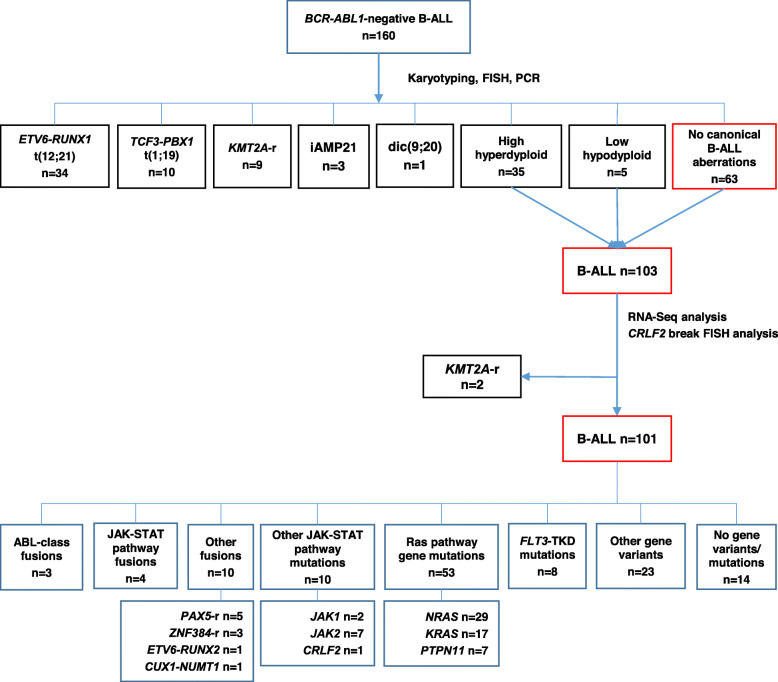
CONSORT diagram of B-ALL study cases. High hyperdiploids, low hypodiploids and cases with no canonical B-ALL alterations, iAMP21 or dic(9;20) were selected for RNA-Seq and FISH (*CRLF2* gene break) analysis. After RNA-Seq data analysis, two cases with *KMT2A* gene rearrangements were excluded. A total of 101 B-ALL patients were screened for non-canonical B-ALL alterations. The number of *FLT3*-TKD, other JAK-STAT and Ras pathway gene mutations corresponds to the total number of specific alterations in sequenced cases

G-banding/SNP-Array, FISH and standard RT-PCR methods [25] were used to identify canonical B-ALL genomic alterations at diagnosis. In order to detect non-canonical B-ALL genomic lesions, we performed RNA-Seq and *CRLF2* gene break FISH analyses in B-ALL cases lacking canonical gene fusions or dic(9:20)/iAMP21 aberrations (Fig. 1).

In total, 99/160 (61.9%) cases harbored at least one canonical B-ALL genomic alteration (Table 2, Fig. 1). High hyperdiploidy was identified in 35/160 (21.9%) patients. Low hypodiploidy was present in 5/160 (3.1%) cases*. ETV6-RUNX1* fusions were detected in 34/160 (21.3%) cases and were more common among children than adults (33 children vs. 1 adult, *p* = 0.001). In contrast*, KMT2A* gene rearrangements were detected in 11/160 (6.9%) B-ALL cases (4 children vs. 7 adults). Importantly, RNA-Seq analysis identified two additional *KMT2A* gene rearrangements (del(11q23)/*KMT2A*-*CBL*, del(11q23)/*KMT2A*-*ATP5L*) that had been missed by FISH. iAMP21 aberration was exclusive to the pediatric group (*n* = 3, 1.9%) while dic(9;20) was present in 1/160 (0.6%) adult patient.

**Table 2:** Genomic subgroups of canonical and non-canonical B-ALL alterations in study patients

	Genomic alterations	Pediatric group (***n*** = 122)	Adult group (***n*** = 38)	Total cohort (***n*** = 160)	***p***-Value (Chi-Square)
**Canonical alterations**	**t(12;21)/*ETV6-RUNX1***	33 (27.1%)	1 (2.6%)	34 (21.3%)	< 0.001
	**t(1;19)/*TCF3-PBX1***	7 (5.7%)	3 (7.9%)	10 (6.3%)	0.701
	**11q23/*KMT2A*** **gene rearrangements**	4 (3.3%)	7 (18.4%)	11 (6.9%)	0.004
	**iAMP21**	3 (2.5%)	0	3 (1.9%)	0.331
	**dic(9;20)**	0	1 (2.6%)	1 (0.6%)	0.071
	**High hyperdiploidy**	32 (26.2%)	3 (7.9%)	35 (21.9%)	0.030
	**Low hypodiploidy**	2 (1.6%)	3 (7.9%)	5 (3.1%)	0.086
	No canonical alterations	41 (33.6%)	20 (52.7%)	61 (38.1%)	
Non-canonical alterations		***n*** **= 75**	***n*** **= 26**	***n = 101***	
	ABL-class fusions	0	3 (11.5%)	3 (3%)	0.003
	JAK-STAT pathway fusions	2 (2.7%)	2 (7.7%)	4 (4%)	0.262
	Other fusions^a^	10 (13.3%)	0	10 (9.9%)	0.050
	JAK-STAT pathway mutations	6 (8%)	4 (15.4%)	10 (9.9%)	0.182
	Ras pathway mutations	39 (52%)	14 (53.8%)	53 (52.5%)	0.772
	*FLT3*-TKD mutations	7 (9.3%)	1 (3.8%)	8 (7.9%)	0.378

^a^PAX5-NCOA5, PAX5-ETV6, PAX5-FOXP1, PAX5-NOL4L, PAX5-GREB1L, EP300-ZNF384, TCF3-ZNF384, ETV6-RUNX2, CUX1-NUTM1

After the exclusion of two patients with *KMT2A* gene rearrangements identified by RNA-Seq, remaining cases without canonical B-ALL gene fusions and cases with high hyperdiploidy or low hypodiploidy (*n* = 101) were selected for RNA-Seq and *CRLF2* gene break FISH analysis to identify other kinase and cytokine receptor activating lesions (Fig. 1).

**Fig. 2:**
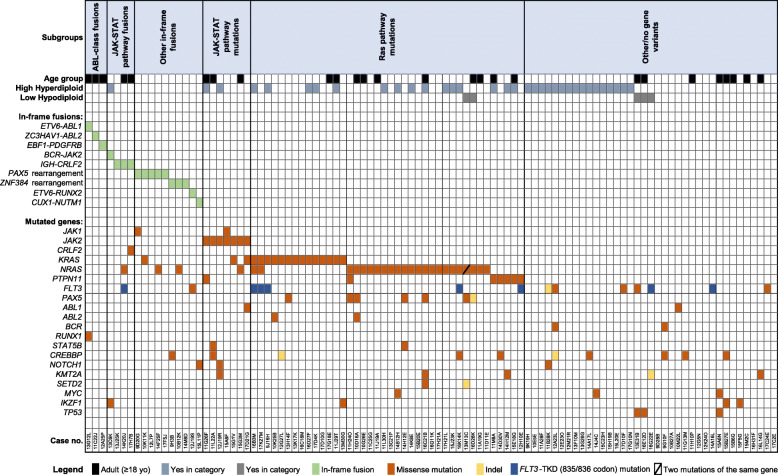
Gene fusions and mutations identified by the RNA-Seq and FISH methods. The cohort is divided into patients with ABL-class fusions, JAK-STAT pathway fusions, other JAK-STAT–activating mutations, Ras pathway mutations, and cases with other or no gene variants

In-frame fusions of ABL-class genes were detected in 3/101 (3%) patients (Table 2; Fig. 1, Fig. 2). One case had t(9;12)/*ETV6-ABL1* fusion previously reported in both lymphoid and myeloid leukemias [26]. The remaining two cases harbored t(1;7)/*ZC3HAV1-ABL2* and t(5;5)/*EBF1*-*PDGFRB* fusions. All ABL-class fusions were exclusive to adults (*p* = 0.003).

JAK-STAT pathway fusions were identified in 4/101 (4%) cases. ABL-class and JAK-STAT pathway fusions were mutually exclusive. RNA-Seq identified t(9;22)/*BCR-JAK2* fusion in one pediatric patient. FISH analysis revealed three cases with t(Y;14) (X;14)/*IGH-CRLF2* gene fusion, of which two were adults. *BCR*-*JAK2* fusion was detected in one case with high hyperdiploidy which further confirms that JAK-STAT pathway gene fusions can be detected in this entity. Both adult cases with *IGH-CRLF2* fusions had additional gene mutations. One *IGH-CRLF2*-positive case harbored *FLT3*-TKD^D835Y^ and *NRAS*^G13R^ gene mutations while another case had *CRLF2*^F232C^ gene mutation. RNA-seq and FISH analysis detected no *P2RY8-CRLF2* fusions in our study patients. SNP Array analysis was available for 81/101 (80.2%) of the sequenced B-ALL patients. No deletions of PAR1 region were identified in any of the cases confirming the absence of *P2RY8-CRLF2* fusion.

We identified other gene fusions in 10/101 (9.9%) pediatric B-ALL cases. Five cases had *PAX5* gene rearrangements: t(9;18)/*PAX5*-*GREB1L,* t(9;20)/*PAX5-NCOA5,* dic(9;12)/*PAX5-ETV6,* dic(3;9)/*PAX5-FOXP1* and dic(9;20)/*PAX5-NOL4L*. One case with *PAX5-GREB1L* fusion had an additional *JAK1*^F838V^ mutation, while in the other two *PAX5*-rearranged cases *KRAS*^G12V^ and *NRAS*^G12C^ mutations were identified. Three cases had fusions involving *ZNF384* gene (*EP300-ZNF384*, *n* = 2*; TCF3-ZNF384*, *n* = 1) of which the *TCF3-ZNF384* positive case had an additional *NRAS*^G13R^ mutation. Though *ETV6*-*RUNX1* is the most common alteration in childhood B-ALL, data analysis revealed a novel fusion of *ETV6* and *RUNX2* genes in one pediatric patient while another patient harbored a t(7;15)/*CUX1-NUMT1* fusion*.*

#### Gene mutation analysis

Gene mutation analysis of RNA-Seq data revealed ten other JAK-STAT pathway mutations in a total of 9/101 (8.9%) cases: *JAK1* (*n* = 2), *JAK2* (*n* = 7), *CRLF2* (*n* = 1) (Table 2; Fig. 2, Additional file: Table S2). One case had both *JAK1* and *JAK2* gene mutations in the same sample. In all cases, protein kinase 1 and kinase 2 domains of *JAK1* and *JAK2* genes were affected. Four *JAK2*-mutated cases were co-mutated with Ras pathway genes (*KRAS* n = 2; *NRAS* n = 1; *PTPN11* n = 1).

A total of fifty-three Ras pathway gene point mutations were detected in 48/101 (47.5%) cases (Table 2; Fig. 2; Additional file 1: Table S2). *NRAS* gene mutations were the most common (*n* = 29) while the incidence of *KRAS* (*n* = 17) and *PTPN11* (*n* = 7) gene mutations was lower. One case had mutations in *NRAS* and *PTPN11* genes while another case had two different *NRAS* gene mutations (G12S and G12A) in the same sample. Two other cases had both *KRAS* and *NRAS* gene mutations in the same sample. Ras pathway mutations were detected in 16/35 (45.7%) high hyperdyploid and 2/5 (40%) low hypodiploidy cases.

*FLT3*-TKD activating mutations of codons D835 (*n* = 5) and I836 (*n* = 3) were found in 8/101 (7.9%) cases. Most of the *FLT3*-TKD positive cases (75%) were present with Ras pathway gene mutations. Other *FLT3* gene kinase domain point mutations were present in 5/101 (5%) patients (Fig. 2).

Other gene mutations were identified in 23/101 (22.8%) patients. Mutations in *FLT3* (*n* = 7), *CREBBP* (*n* = 5) and *TP53* (*n* = 3) genes were the most recurrent in this group of patients. RNA-Seq and FISH methods failed to identify ALL-related gene fusions and mutations in 14/101 (13.9%) cases.

### Clinical outcome of patients with ABL-class or JAK-STAT fusions

**Fig. 3:**
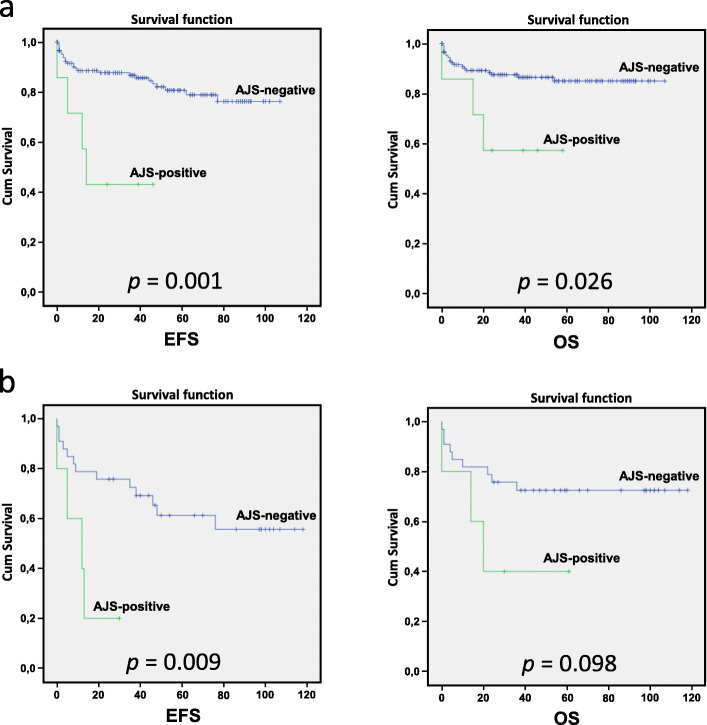
Event-free survival (EFS) and overall survival (OS) in months in ABL-class or JAK-STAT pathway fusion (AJS)-positive vs. AJS-negative patients. **a**, all B-ALL patients (7 AJS-positive vs 153 AJS-negative pts); **b**, adult B-ALL patients (5 AJS-positive vs 33 AJS-negative pts)

Five of 26 (19.2%) adult and two of 75 (2.7%) pediatric B-ALL cases without canonical B-ALL alterations (5/38 (13.2%) adult and 2/122 (1.6%) pediatric *BCR*-*ABL1*-negative B-ALL cases) were positive for either ABL-class or JAK-STAT pathway fusions (AJS-positive group) (Table 3). Overall, both EFS and OS were worse in the AJS-positive group vs the AJS-negative (*n* = 153) (Fig. 3a).

**Table 3:** Genetic features and clinical outcome of ABL-class or JAK-STAT pathway fusion (AJS)-positive patients

Case no.	Age group	In-frame gene fusion	WBC	DEX / PREDNI	MRD D15	MRD D29	MRD D79	Risk Group	alloSCT (Y/N)	Event (Y/N)	Comment
Case 1	Adult	*ETV6-ABL1*	270	DEX	─	─	─	─	N	Y	Induction death
Case 2	Adult	*ZC3HAV1-ABL2*	72	PREDNI	30.5%	6.5%	0.01%	IR	Y	Y	Death of sepsis after alloSCT in CR1
Case 3	Adult	*EBF1-PDGFRB*	258	DEX	45.3%	0.93% (POST A1)	0.7% (POST B1)	HR-SCT	Y	Y	Relapse after alloSCT and death
Case 4	Adult	*IGH-CRLF2*	4	PREDNI	11.6%	20.3%	3.4%	HR-SCT	Y	Y	Relapse after alloSCT and alive in CR2
Case 5	Adult	*IGH-CRLF2*	104	DEX	44.3%	8%	1.65%	HR-SCT	Y	N	Alive after alloSCT in CR1
Case 6	Pediatric	*IGH-CRLF2*	35	PREDNI	0.22%	< 0.1%	0.3%	HR-SCT	Y	N	Alive after alloSCT in CR1
Case 7	Pediatric	*BCR-JAK2*	5	PREDNI	1.8%	0.33%	< 0.1%	IR	N	N	Alive in CR1

*WBC* White blood cells, *DEX* Dexamethasone, *PREDNI* Prednisolone, *D15* Day 15, *D29* Day 29, *D79* Day 79, *MRD* Minimal residual disease, *alloSCT* Allogenic stem cell transplant, *CR1* First clinical remission, *CR2* Second clinical remission

We further compared the outcome of AJS positive (*n* = 5) vs. AJS negative (*n* = 33) B-ALL cases in adults since the number of AJS-positive pediatric cases was too small for detailed analysis. Four AJS-positive adult patients were evaluated for residual disease on induction day 15 (one patient died during induction). All four AJS-positive patients (100%) had ≥5% blasts on day 15 compared to 9 (29%) AJS-negative patients (*p* = 0.019) (Table 4). After day 15, one patient was assigned to block treatment; therefore, three patients had MRD data available on day 29. MRD levels on days 29 and 79 were higher in the AJS-positive group compared to the AJS-negative group. As a result of a poor MRD response, more AJS-positive than AJS-negative adult patients had induction failure or were assigned to HR/HR-SCT group (4 (80.0%) vs 10 (30.3%), respectively, *p* = 0.052).

**Table 4:** Minimal residual disease (MRD) of AJS-positive vs. AJS-negative adult patients on days 15, 29 and 79

Timepoint	MRD	AJS-positive (***n*** = 5)	AJS-negative (***n*** = 33)	***p***-Value (Chi-Square)
D15	< 5%	0 (0%)	22 (71%)	0.019
	≥5%	4 (100%) 1 missing	9 (29%) 2 missing	
D29	< 5%	0 (0%)	30 (96.8%)	< 0.001
	≥5%	3 (100%) 2 missing	1 (3.2%) 2 missing	
D29	< 0.1%	0 (0%)	21 (67.7%)	0.011
	≥0.1%	3 (100%) 2 missing	10 (32.3%) 2 missing	
D79	< 0.1%	2 (50%)	26 (89.7%)	0.120
	≥0.1%	2 (50%) 1 missing	3 (10.3%) 4 missing	

*AJS* ABL-class or JAK-STAT pathway fusions, *D15* Day 15, *D29* Day 29, *D79* Day 79m, *MRD* Minimal residual disease

The median observation time in adults was 39 months. The 75th percentile EFS was 5 vs. 35 months (*p* = 0.009) and OS was 14 vs. 36 months (*p* = 0.098) in AJS-positive vs. AJS-negative adults, respectively (Fig. 3b). Since all patients were assigned to different risk groups according to their canonical B-ALL alterations and MRD response, we performed a multivariate analysis of risk-group assignment and AJS-positivity on the outcome in adults. In multivariate analysis, having ABL-class or JAK-STAT pathway fusions was an independent risk factor for worse EFS (*p* = 0.046) (Table 5).

**Table 5:** Multivariate analysis of EFS and OS of MRD-assigned risk and AJS-positive groups in adults

Risk Groups	EFS		OS	
	Hazard Ratio	***p***-Values	Hazard Ratio	***p***-Values
SR	reference	–	reference	–
IR	0.885 (0.220–3.570)	0.846	2.219 (0.257–19.136)	0.468
HR + HR-SCT	0.753 (0.146–3.875)	0.734	1.568 (0.134–18.298)	0.720
AJS-positive	5.545 (1.057–29.090)	0.046	2.783 (0.436–17.881)	0.278

*SR* Standard risk, *IR* Intermediate risk, *HR* High risk, *HR-SCT* High risk–stem cell transplant groups, *AJS* ABL-class or JAK-STAT pathway fusions, *EFS* Event-free survival, *OS* Overall survival, *MRD* Minimal residual disease

## Discussion

We present the first Baltic European population-based study of genomic alterations among pediatric and adult *BCR*-*ABL1*-negative B-ALL patients. All patients were uniformly treated according to the NOPHO ALL-2008 protocol with risk stratification according to both the canonical B-ALL genomic lesions and minimal residual disease. We selected targeted RNA-Seq and FISH methods for the detection of known and novel gene rearrangements of ABL-class, JAK-STAT pathway genes and other kinase alterations.

In our study, ABL-class fusions were identified in 3/101 (3%) B-ALL cases without canonical B-ALL alterations or 3/160 (1.9%) *BCR*-*ABL1*-negative B-ALL cases. All positive cases were adults (3/38, 7.9%) (Fig. 2). In comparison, in the Dutch/German cohort 9 of 153 (5.9%) pediatric B-ALL patients without canonical B-ALL alterations harbored ABL-class fusions [14]. Another European study by Zaliova et al. revealed only 1/75 (1.3%) pediatric B-ALL case with *ABL1* gene rearrangement [27] while ABL-class fusions were present in 40/1389 (2.9%) high-risk pediatric B-ALL patients in an US Children’s Oncology Group study [13]. Heatley at el. performed targeted RNA-Seq in 63 adolescent/young adults (16–39 yo) and 63 adults (40–88 yo) with *BCR-ALB1*-negative B-ALL [28]. ABL-class fusions were found in 4/126 (3.2%) of these B-ALL cases. A study by Garrido et al. used the FISH method to analyze 39 adult B-ALL patients negative for the *BCR*-*ABL1* and *KMT2A* gene rearrangements and identified 3/39 (7.7%) cases with ABL-class fusions (*ABL1 n* = 2; *CSF1R n* = 1) [29]. In a similar study from a UKALL14 clinical trial, ABL-class abnormalities were present in 6/648 (< 1%) of B-ALL patients [30]. The incidence of ABL-class fusions of 7.9% in our adult patient cohort was largely in line with published adult studies. Conversely, we did not detect ABL-class fusions in our pediatric B-ALL patients.

We identified JAK-STAT pathway fusions in 4/101 (4%) cases without canonical B-ALL alterations. JAK-STAT pathway fusions occur in approximately 3–5% of childhood and in up to 15% of adult B-ALL [3]. A study by Heatley et al. showed the incidence rate in adults of 15.9% [28]; however, another study revealed that only 5.1% of adult B-ALL had JAK-STAT pathway fusions [30]. Similarly, our data indicate a lower frequency of JAK-STAT pathway fusions in both adult and pediatric Lithuanian B-ALL patients.

Overall, the frequency of *CRLF2* rearrangements in B-ALL is approximately 5% and the rate gets higher in cases without canonical B-ALL alterations (10–30%) and in patients with Down syndrome (> 50%) [16, 27, 28, 31, 32]. In our study, *IGH-CRLF2* fusions were identified in 3/101 (2.9%) B-ALL cases without canonical B-ALL alterations or in 3/160 (1.9%) *BCR*-*ABL1*-negative B-ALL cases. Notably, we did not detect any *P2RY8-CRLF2* gene fusions in our cohort by either RNA-Seq or *CRLF2* gene break FISH. In comparison, a Swedish study identified *CRLF2* gene rearrangements in 16 of 189 (8.5%) pediatric *BCR-ABL*-negative B-ALL patients, of which *P2RY8-CRLF2* fusion was the most common (12/16, 75%) [16]. *P2RY8-CRLF2* gene fusion is the result of deletion of PAR1 region in either X or Y chromosomes. To further confirm the absence of *P2RY8-CRLF2* fusion, we used SNP Array data of 81/101 (80.2%) sequenced patients and did not detect any deletions in the corresponding PAR1 region. In addition, another study has shown that *P2RY8-CRLF2* fusion can be identified using the RNA-Seq method and an analysis algorithm similar to ours [33], making it unlikely that *P2RY8-CRLF2* fusion was missed due to technical reasons in our study patients. *CRLF2* gene rearrangements are common in populations with Hispanic ancestry and are associated with increased risk of relapse in both children and adults [17, 34–36]. Population differences may explain a lower frequency of *IGH*-*CRLF2* and the absence of *P2RY8-CRLF2* gene fusions in our cohort. In other B-ALL studies, approximately half of *CRLF2*-rearranged patients also harbored additional *JAK1* or *JAK2* gene mutations [13], however our positive cases had no such mutations. Of note, one case had *FLT3*-TKD^D835Y^ and *NRAS*^G13R^ gene mutations and another case was positive for a *CRLF2*^F232C^ activating mutation. *CRLF2*^F232C^ gene mutation is known to promote constitutive dimerization and cytokine-independent growth which results in gene overexpression in a similar manner as with *CRLF2* gene rearrangement [37].

Other gene fusions were found in 10/101 (9.9%) patients and were exclusive to the pediatric group (Fig. 2). RNA-Seq data were used for gene mutation analysis with a caveat that only expressed sequence variants of targeted genes which could be evaluated. In most cases, the coverage of particular mutations calculated from RNA-Seq data was inadequate to accurately determine their frequencies in the sample. Therefore, we did not evaluate mutation-based clonality/subclonality. In our study, other gene fusions and point mutations in *FLT3,* JAK-STAT and Ras pathway genes had no prognostic significance on either EFS or OS among all age groups.

RNA-Seq analysis revealed two adult B-ALL cases with *KMT2A* gene rearrangements that had been missed by FISH analysis at diagnosis. Both samples had blast counts of > 90%. We re-ran the FISH analysis; however, the secondary results were also negative. Notably, both fusions were formed as a result of the aberrations of the 11q23 chromosome region. The close proximity of fused genes and relatively small size of chromosome aberrations could have potentially caused false negative FISH results. Patients with *KMT2A* gene rearrangements have lower survival rates and generally require therapy intensification in first remission [2]. *KMT2A-ATP5L* fusion was also recently described as an unfavorable prognostic marker in young adults with Ph-like ALL [38]. Our findings demonstrate the additional advantage of RNA-Seq diagnostic method in B-ALL patients who are negative for canonical B-ALL fusions by FISH analysis.

Ph-like B-ALL has been associated with a worse outcome in several studies [18, 19, 39]. Roberts with colleagues [21] performed a gene expression profiling of 344 B-ALL patients and found that 40/344 (11.6%) children with Ph-like B-ALL had higher MRD values on both day 19 and at the end of induction compared to non-Ph-like B-ALL patients. However, the EFS and OS were not different in both groups due to MRD-risk directed treatment of the Total Therapy XV protocol [21]. Ph-like B-ALL adult patients were noted to have a lower probability of achieving molecular complete remission, and had a lower probability of continuous complete remission and OS in the GMALL studies 06/99 and 07/03 [20]. Only limited MRD-directed treatment was applied in GMALL 07/03 study with persistently MRD positive standard-risk patients having an option of SCT [40]. In another study of patients entering different study protocols, Ph-like B-ALL was associated with a worse outcome in young but not older adults [41]. Likewise, lower EFS and OS were noted in adult Ph-like B-ALL patients in the MD Anderson studies [19]. In a CALGB10403 study of young (18–40 year-old) adults treated according to a pediatric high-risk non-MRD directed protocol, patients with Ph-like fusions had a 3-year EFS of 42% in contrast to 69% for those without these fusions and patients with the Ph-like B-ALL signature were less likely to have the negative MRD compared to patients without this signature [42].

There are several important differences in our study. First, all patients were treated according to the MRD-risk directed NOPHO ALL-2008 protocol and patients with a poor MRD response were assigned to therapy intensification with or without allogeneic SCT [2]. Second, we studied the ABL-class or JAK-STAT pathway fusion-positive patients only who mostly, but not exclusively, clustered within a Ph-like group [18]. Nevertheless, we found that patients with ABL-class or JAK-STAT pathway fusions (AJS-positive group) were more likely to have a poor MRD response compared to patients without these fusions (AJS-negative group) and thus were more likely to be assigned to higher risk groups resulting in therapy intensification. In our study, AJS-positive adult patients had a lower EFS and a trend for lower OS. In multivariate analysis, AJS-positivity was a risk factor for worse EFS (*p* = 0.046) but not OS (*p* = 0.278) in adults (Table 5).

Our results are supported by pediatric B-ALL studies focusing on a cytogenetic analysis of ABL-class and/or JAK-STAT pathway fusions. A study of UKALL2003 trial patients by O’Connor et al. [11] examined pediatric ALL patients with induction failure (leukemic blasts > 5% and/or MRD ≥5%) revealing a high rate of ABL-class (> 30%) or CRLF2 (11%) rearrangements. Similarly, in a study by Cario et al., 46 pediatric B-ALL patients with ABL-class fusions treated according to AIEOP-BFM protocols showed high rates of MRD (≥5 × 10–4) positivity (71.4 and 51.2% after induction and consolidation, respectively) [43]. We observed similar poor MRD responses in our ABL-class / JAK-STAT fusion-positive adult patients despite MRD directed treatment. Importantly, ABL-class positive patients who received a TKI as part of their UKALL2011 protocol first remission treatment showed a lower relapse rate [12].

Our study has limitations. Though the NOPHO 2008 protocol included children and adults from Nordic, Baltic (Lithuania and Estonia) countries and Iceland, we had access to and studied Lithuanian patients only. The number of ABL-class or JAK-STAT pathway fusion-positive patients was low and a few events occurring by chance could have significantly affected the statistics of clinical outcome. As already mentioned, our results are not directly comparable to the previous studies of Ph-like B-ALL. Nevertheless, the results of our study are clinically relevant. We report the results of a relatively large and homogenous group of pediatric and adult patients coming from the Baltic part of Europe and show that the incidence of ABL-class or JAK-STAT pathway fusions in this geographic area is lower as compared to the US or other European regions [11–14, 16, 18, 30]. Our results suggest that AJS-positive group patients have a poor MRD response and MRD-risk directed treatment may not be sufficient to overcome the adverse effect of AJS-positivity in adults. Importantly, clinical studies have shown that specific ABL-class fusions can be successfully targeted with tyrosine kinase inhibitors such as dasatinib or imatinib even in refractory B-ALL [7, 8, 10, 12, 43]. Arguably, the detection of ABL-class or JAK-STAT pathway fusions could be reserved for poor B-ALL MRD responders. This could have significant financial and logistical implications since only a limited number of B-ALL patients will need to be tested for ABL-class or JAK-STAT pathway fusions.

## Conclusions

ABL-class and JAK-STAT pathway fusions are uncommon in the population-based cohort of Lithuanian *BCR*-*ABL1*-negative B-ALL patients. Specifically, *P2RY8-CRLF2* gene fusion was not detected using three different genome interrogation methods. The poor MRD response and an inferior clinical outcome in patients harboring either ABL-class or JAK-STAT pathway fusions should be confirmed in future prospective studies.

## Supplementary Information

**Additional file 1: Table S1, Table S2**.

## Data Availability

The datasets used during the current study are available from the corresponding author on reasonable request.

## Abbreviations

ALL: Acute lymphoblastic leukemia
EFS: Event-free survival
FISH: Fluorescence in situ hybridization
GMALL: German multicenter study group for adult ALL
HR: High risk
IR: Intermediate risk
JAK-STAT: Janus kinase-signal transducers and activators of transcription
MRD: Minimal residual disease
NOPHO: Nordic Society for Pediatric Hematology and Oncology
OS: Overall survival
PCR: Polymerase chain reaction
SCT: Stem cell transplant
SNP: Single nucleotide polymorphism
SR: Standard risk
TKI: Tyrosine kinase inhibitor
