# Supplementary material for: Low incidence of ABL-class and JAK-STAT signaling pathway alterations in uniformly treated pediatric and adult B-cell acute lymphoblastic leukemia patients using MRD risk-directed approach – a population-based study
Source: BMC Cancer. 2021 Mar 29;21:326. doi: 10.1186/s12885-020-07781-6 (PMC8006339; doi:10.1186/s12885-020-07781-6)
Supplement: Supplementary file 1 — Additional file 1: Table S1, Table S2. [file 12885_2020_7781_MOESM1_ESM.docx]

**Table S1.** PCR primer pairs used to confirm gene fusions detected by RNA-Seq. *ABL1*, *ABL2, PDGFRB, JAK2* and *CRLF2* gene rearrangements were confirmed by FISH.

| **Case** | **Gene fusion** | **Forward primer 5’ ‒ 3’** | **Reverse primer 5’ ‒ 3’** |
| --- | --- | --- | --- |
| 9D20G | *PAX5-GREB1L* | ACAGGGCAGCTACTCAGCAC | GTGTGGGCTGGAGTGGAG |
| 10K11K | *PAX5-NCOA5* | GGTACCCTCCACACGTCC | CTGGGTCTTGATGGAGCCG |
| 12L7P | *PAX5-ETV6* | GTGCCTAGCGTCAGTTCCAT | GTGTTGCTGTCAATTGGCCTTAA |
| 14F25F | *PAX5-FOXP1* | CAGATGCGGGGAGACTTGTT | GCTGCTGAAGAAGGAGCTGT |
| 17F5J | *PAX5-NOL4L* | CCTCGGTGAGCACGGATT | CTGGTGCTGGTGCTGGAG |
| 9H3B,14M8D | *EP300-ZNF384* | TCAGATGCCGACACAACCC | GGGGATAGAAGGCCAGAAGT |
| 10B12K | *TCF3-ZNF384* | CCCGGATCACTCAAGCAATA | GGGGATAGAAGGCCAGAAGT |
| 12J19S | *ETV6-RUNX2* | CTGGCTTACATGAACCACATCAT | GGCTGCAGGCTGCTGGA |
| 15L11P | *CUX1-NUTM1* | TCCAAGAATTAGTAGCCATGTCC | GAAGTGCAGGGGATGGAGA |
| 14B14C | *KMT2A*-*CBL* | TGGGAGGCTTAGGAATCTTGA | GGCAGATGAGGAAGGTTTGAT |
| 17A9B | *KMT2A*-*ATP5L* | AAGTGGCTCCCCGCCCAA | CTCCGACATAAAACCACATCAAC |

**Table S2**. Identified JAK-STAT and Ras pathway gene point mutations in B-ALL study cases.

| **Signaling pathway** | **Mutated gene** | **Transcript (HGVSp)** | **Mutation** | **Case** |
| --- | --- | --- | --- | --- |
| JAK-STAT pathway | *JAK1* | NP_002218.2 | p.E668D | 15A8F |
|  |  |  | p.F838V | 9D20G |
|  | *JAK2* | NP_004963.1 | p.R564L | 15A8F |
|  |  |  | p.G571S | 11L22A |
|  |  |  | p.R683G | 17D21G |
|  |  |  | p.D873N | 12J19R |
|  |  |  | p.R1063H | 11G26F, 15G1V, 15G3M |
|  | *CRLF2* | NP_071431.2 | p.F232C | 17H7B |
| Ras pathway | *KRAS* | NP_203524.1 | p.G12D | 17D21G, 16B3M, 12G27L, 17G18E, |
|  |  |  | p.G12V | 10K11K, 17K27M, 13H14F |
|  |  |  | p.G12S | 15G1V |
|  |  |  | p.G13D | 9J16H, 13K17K, 16C18M, 17D10G, 11J29T, 13M30G |
|  |  |  | p.Q61P | 16D27F |
|  |  |  | p.A146V | 10K25B, 17D4K |
|  | *NRAS* | NP_002515.1 | p.G12A | 13M13C |
|  |  |  | p.G12C | 14F25F, 15B20E |
|  |  |  | p.G12D | 17K27M, 11J19A, 11L30H, 13C21P, 14B12H, 16K14K, 11A19G |
|  |  |  | p.G12V | 11H24O |
|  |  |  | p.G12S | 10D16A, 13M13C |
|  |  |  | p.G13D | 15G3M, 16B3M, 11C25G, 14H12E, 17H21L, 15J23K |
|  |  |  | p.G13R | 14K20J, 10B12K |
|  |  |  | p.Q61H | 10B12K, 14K8E, 11D11E |
|  |  |  | p.Q61K | 16C21B, 17H21A |
|  |  |  | p.Q61R | 10G26E, 16G11K |
|  |  |  | p.A146T | 16D26K |
|  | *PTPN11* | NP_002825.3 | p.D61H | 11H24O |
|  |  |  | p.D61Y | 14D30V |
|  |  |  | p.A72V | 15E18G, 12H10E |
|  |  |  | p.E76G | 11G26F |
|  |  |  | p.E76K | 14H12M |
|  |  |  | p.E139D | 11M8A |
